# *Staphylococcus epidermidis*: A differential trait of the fecal microbiota of breast-fed infants

**DOI:** 10.1186/1471-2180-8-143

**Published:** 2008-09-10

**Authors:** Esther Jiménez, Susana Delgado, Antonio Maldonado, Rebeca Arroyo, Mar Albújar, Natalia García, Manel Jariod, Leonides Fernández, Adolfo Gómez, Juan M Rodríguez

**Affiliations:** 1 Departamento de Nutrición, Bromatología y Tecnología de los Alimentos, Universidad Complutense de Madrid, 28040, Madrid, Spain; 2 Servei de Pediatria, Hospital Universitari Joan XXIII, 43007, Tarragona, Spain

## Abstract

**Background:**

Breast milk is an important source of staphylococci and other bacterial groups to the infant gut. The objective of this work was to analyse the bacterial diversity in feces of breast-fed infants and to compare it with that of formula-fed ones. A total of 23 women and their respective infants (16 breast-fed and 7 formula-fed) participated in the study. The 16 women and their infants provided a sample of breast milk and feces, respectively, at days 7, 14, and 35. The samples were plated onto different culture media. Staphylococcal and enterococcal isolates were submitted to genetic profiling and to a characterization scheme, including detection of potential virulence traits and sensitivity to antibiotics.

**Results:**

The feeding practice had a significant effect on bacterial counts. A total of 1,210 isolates (489 from milk, 531 from breast-fed and 190 from formula-fed infants) were identified. *Staphylococcus epidermidis *was the predominant species in milk and feces of breast-fed infants while it was less prevalent in those of formula fed-infants. *Enterococcus faecalis *was the second predominant bacterial species among the fecal samples provided by the breast-fed infants but it was also present in all the samples from the formula-fed ones. The biofilm-related *icaD *gene and the *mecA *gene were only detected in a low number of the *S. epidermidis *strains. Several enterococcal isolates were also characterized and none of them contained the *cylA *or the *vanABDEG *antibiotic-resistance genes. All were sensitive to vancomycin.

**Conclusion:**

The presence of *S. epidermidis *is a differential trait of the fecal microbiota of breast-fed infants. Globally, the staphyloccal isolates obtained from milk and feces of breast-fed infants contain a low number of virulence determinants and are sensitive to most of the antibiotics tested.

## Background

Although the composition of the human intestinal microbiota is a major factor in the health status of both adults and infants, the process of initial colonization of the neonatal gut and the origin of the first colonizers are aspects that remain unclear.

Although the colonization pattern seems to be host-specific, this is generally accepted that, initially, the infant gut would contain facultative anaerobes which would create a reduced environment favourable to the establishment of obligate anaerobes, such as *Bacteroides*, *Clostridium *and *Bifidobacterium *species [1].

Traditionally, it has been widely accepted that the development of the gut microbiota starts at birth and is greatly influenced by the type of feeding [1-4]. The bacterial spectrum of breast-fed infants feces is narrower than that of formula-fed ones although, in the formers, the counts of fecal bifidobacteria and lactic acid bacteria are usually notably higher than those found in formula-fed infants [5,6]. Once weaning starts, differences between breast-fed and formula-fed infants disappear rapidly and the gut ecosystem evolves into a stable host-specific community predominated by obligate anaerobes [1].

Human milk is a major factor in the initiation and development of neonatal gut microbiota, not only because it contains prebiotic substances that promote the growth of selected bacterial groups in the infant gut [7], but also because this substrate represents a continuous source of microorganisms to the infant gut during several weeks after birth [8,9]. The presence of a few predominant bacterial species in breast milk [10] may explain why gut microbiota of breast-fed infants is composed of a narrow spectrum of species, and a more diverse microbiota develops only after weaning.

However, few studies on the factors influencing the composition of the intestinal microbiota in early infancy have had into account the influence of the bacteria naturally present in human milk [11]. Staphylococci, and particularly *Staphylococcus epidermidis*, seems to be the most predominant bacteria both in fresh and stored human milk [9,10], but paradoxically they have received a marginal attention regarding their role in the early colonization of the infant gut. Additionally, the few studies that report the detection of staphylococci in neonatal and infant feces are controversial since their presence has been rated from low [1] to high [12]. In this context, the objective of this study was to compare the bacterial diversity of breast milk, feces of breast-fed infants and feces of formula-fed ones by culture-based methods, with particular attention to those species belonging to the Genus *Staphylococcus*. Additionally, we investigated the role of breast milk as a source of staphylococci to the infant gut and characterized the *S. epidermidis *strains isolated from the feces of the breast-fed infants. Finally, since enterococci seems to be another predominant bacterial group in the gut of both breast- and formula-fed infants, the characterization of the enterococcal strains isolated from infant feces constituted another objective of this study.

## Results

### Bacterial counts in fecal and milk samples

Inoculation of suitable dilutions of the different fecal samples (breast- or formula-fed 7-, 14-, or 35-day-old infants) led to bacterial growth in all the culture media tested. Globally, the values oscillated between 9.25 and 10.93 log_10 _CFU/g (Table 1). A two-way ANOVA, with feeding practice and infant age as factors, revealed that the feeding practice had a significant effect on bacterial counts in infant feces (F-value = 10.11, P = 0.0045) but the influence of infant age was irrelevant. The mean bacterial counts in the feces of breast-fed infants were almost 1 log_10 _CFU/g lower than the values corresponding to feces of the formula-fed ones as revealed by Duncan's test (Table 1). A second analysis with the culture medium used for enumeration and the feeding practice as factors in a two-way ANOVA showed important influence of the culture media (F-value = 11.09, P < 0.0001) on the fecal bacterial counts (Table 1). Aerobic bacterial counts (9.25 and 9.54 log_10 _CFU/g obtained in CNA and VRBA media, respectively) were significantly lower than anaerobic bacterial counts (10.93 and 10.67 log_10 _CFU/g obtained in WCh and MRS media, respectively). Mean bacterial counts on BHI medium had intermediate values. Furthermore, in this second analysis, the interaction between the culture medium employed and the feeding practice was also significant (F-value = 3.32, P = 0.0141), suggesting a differential influence of the feeding practice on the bacterial groups present in infant feces (Table 1).

**Table 1:** Effects and interactions of feeding practice and infant age or culture media used for enumeration on bacterial counts in infant feces, as determined by repeated measures two-way ANOVA

Effect or interaction	DF	F value	Pr > F^a^	Duncan's grouping^b^	N	Group	Mean (log_10 _CFU g^-1^)
Feeding practice	1	10.11	0.0045	A	215	Breast-fed	9.87
				B	105	Formula-fed	10.71
Infant age	2	0.18	0.8343	A	115	7-day old	10.16
				A	105	14-day old	10.16
				A	100	35-day old	10.12
Feeding practice × infant age	2	1.12	0.3360				

Feeding practice	1	10.18	0.0044	A	215	Breast-fed	9.87
				B	105	Formula-fed	10.71
Culture medium	4	11.09	< 0.0001	A	64	CNA	9.25
				A	64	VRBA	9.54
				B	64	BHI	10.34
				BC	64	MRS	10.67
				C	64	WCh	10.93
Feeding practice × culture medium	4	3.32	0.0141				

^a^Probability value F test: significant when P < 0.05.

^b^Duncan's tests: groups within the same effect with the same letter do not differ significantly (P < 0.05).

N = 320

A more detailed comparison of the mean bacterial counts in feces from breast-fed and formula-fed infants considering the results obtained in the different culture media for each infant age revealed significant differences (P < 0.05) in the bacterial counts corresponding to the culture media that were incubated aerobically (BHI, VRBA and CNA) for the samples obtained from 1- and 2-week old infants, but not after 5 weeks (Table 2). In contrast, no significant differences were observed on bacterial counts at any infant age assayed in those media (WCh and MRS) that were incubated anaerobically.

**Table 2:** Bacterial counts expressed as the mean log_10 _CFU g^-1 ^(SD) in feces of the breast-fed (n = 16) and formula-fed (n = 7) infants

Medium	Week	Breast-fed infants	Formula-fed infants	P-value*
BHI	1	9.93 (0.95)	11.42 (0.68)	0.0078
	2	9.92 (0.51)	11.05 (0.39)	0.0001
	5	10.06 (1.01)	10.83 (0.80)	NS
VRBA	1	9.17 (1.07)	10.61 (1.09)	0.0415
	2	9.24 (0.93)	10.62 (0.09)	0.0011
	5	9.49 (1.35)	10.04 (0.70)	NS
CNA	1	9.20 (0.91)	10.66 (0.73)	0.0143
	2	9.08 (1.04)	10.46 (0.27)	0.0030
	5	8.52 (1.05)	9.74 (0.84)	NS
WCh	1	10.76 (0.91)	11.25 (0.97)	NS
	2	10.42 (0.99)	11.12 (0.20)	NS
	5	11.01 (0.55)	10.85 (0.51)	NS
MRS	1	10.51 (0.98)	10.83 (1.04)	NS
	2	10.22 (1.03)	10.61 (0.27)	NS
	5	10.57 (0.59)	10.51 (0.52)	NS

Statistical significance between the breast-fed group and the formula-fed group (Student's *t*-test). NS, not significant difference.

The average bacterial count in milk samples was 4.16 log_10 _CFU/ml when they were inoculated in BHI, CNA, WCh and MRS media (Table 3). The CNA means decreased significantly (P = 0.031, Student's *t*-test) from day 7 to 35 (from 4.22 to 3.69 log_10 _CFU/ml). In the rest of the media, there were no statistically significant differences between the mean bacterial counts through the period studied. When the samples were inoculated in VRBA agar, no colonies could be isolated in 9, 8 and 6 milk samples at days 7, 14 and 35, respectively (Table 3). Globally, VRBA bacterial growth was not detected in any of the three samples provided by 4 of the women that participated in the study. The mean of the VRBA in the rest of the samples oscillated between 4.42 and 4.80 log CFU/ml. A relationship (P < 0.070) could be observed between the bacterial counts from breast milk and from infant feces in CNA (weeks 1, 2 and 5), MRS (week 1) and WCh (week 1).

**Table 3:** Bacterial counts expressed as the mean log_10 _CFU/g (SD) from breast milk of the mothers that participated in the study

Medium	Week 1 (n = 16)	Week 2 (n = 13)	Week 5 (n = 14)	F^a^
BHI	4.63 (0.89)	4.58 (1.44)	4.22 (1.08)	0.72
VRBA	ND^b ^(n = 9)	ND (n = 8)	ND (n = 6)	-
	4.80 (1.35) (n = 7)	4.74 (1.21) (n = 5)	4.42 (0.91) (n = 8)	-
CNA	4.22 (0.55)	4.03 (0.85)	3.69 (0.62)	2.97
WCh	4.22 (0.67)	4.43 (0.86)	4.27 (1.20)	1.41
MRS	3.97 (0.70)	3.79 (0.96)	4.14 (1.22)	0.21

^a^F_2,10 _value from a repeated measures ANOVA testing the effect of sampling occasion on bacterial counts of breast milk for each culture media used (p > 0.05)

^b^ND, not detected

### Identification of the isolates

A total of 721 isolates were randomly selected from CNA, BHI, MRS and WCh agar plates corresponding to feces of the breast-fed (531 isolates) and formula-fed (190 isolates) infants. Subsequently, they were identified by classical morphological and biochemical tests, species-specific PCR and/or 16S rDNA sequencing.

*S. epidermidis *was the predominant species in feces of breast-fed infants since it could be isolated from 86.05% of the samples (Table 4). In this group, a slight decrease in the number of *S. epidermidis *positive samples was observed from day 7 (16/14) to day 35 (9/13). In contrast, this species was only present in 13.33% of the samples obtained from formula-fed infants. The difference in the number of *S. epidermidis*-positive samples between breast- and formula-fed infants was statistically significant (P < 0.0001). Presence of *S. aureus *and other *Staphylococcus *species was similar in both groups.

**Table 4:** Bacteria detected in the samples of breast milk and feces of the breast- and formula-fed infants and percentage of samples in which they were detected

		Feces		
			
Microorganism	Milk	Breast-fed infants	Formula-fed infants	P-value^a^
*Staphylococcus epidermidis*	100.00%	86.05%	13.33%	< 0.0001
*Staphylococcus aureus*	16.28%	16.28%	13.33%	NS^b^
Other *Staphylococcus *spp.	16.28%	6.98%	6.67%	NS
*Enterococcus faecalis*	20.93%	53.49%	100.00%	0.0011
*Enterococcus faecium*	ND^c^	2.33%	13.33%	NS
Other *Enterococcus *spp.	4.65%	9.30%	26.67%	NS
*Streptococcus *spp.	27.91%	13.95%	ND	NS
Other Gram-positive bacteria	20.93%	69.77%	33.33%	0.0130
Gram-negative bacteria	46.51%	97.62%	66.67%	0.0008

^a ^Statistical significance between the breast-fed group and the formula-fed group (χ^2 ^test).

^b^NS, not significant (*P *> 0.05).

^c^ND, not detected.

*Enterococcus faecalis *was the second Gram-positive bacterial species more widespread among the samples of the breast-fed group (53.49%). However, since it was present in 100% of the samples from the formula-fed one, there was a significant difference between both groups regarding this microorganism (P = 0.0011). Other enterococcal species were also more widespread among feces of the formula-fed infants but the differences were not statistically different (Table 4). In contrast, streptococci could only be isolated from feces of breast-fed infants. The percentage of samples containing other Gram-positive bacteria (including bifidobacteria) and Gram-negative bacteria was also significantly higher (P = 0.013 and 0.0008, respectively) in samples from breast-fed infants (Table 4).

Globally, 140 isolates (26.36%) from the breast-fed group were identified as *S. epidermidis*, 76 (14.31%) as *Bifidobacterium *sp. (*B. adolescentis*, *B. brevis*, *B. infantis*, *B. bifidum*, *B. longum*, *B. pseudocatenulatum*, *B. dentium*, and *B. angulatum*) and 69 (12.99%) as *E. faecalis *(Table 5). Interestingly, only 3 isolates (1.57%) from the formula-fed group belonged to the *S. epidermidis *species while other 3 isolates (1.57%) were identified as *Bifidobacterium *spp. In contrast, 84 isolates (44.21%) of this group were *E. faecalis *and this percentage increases to 52.63% when having into account all the enterococcal species identified (Table 4). A considerable percentage (31–35%) of isolates from both breast- and formula-fed infants was identified as Gram-negative bacteria although they belonged to a wide spectrum of species (Table 5).

**Table 5:** Identification of the colonies isolated from breast milk and feces from breast- and formula-fed infants

	Milk	Breast-fed infants	Formula-fed infants
Microorganisms	N° colonies (%)	N° colonies (%)	N° colonies (%)

1. Gram-positive bacteria:			
*Staphylococcus epidermidis*	304 (62.16)	140 (26.36)	3 (1.57)
Other staphylococci	24 (4.91)	21 (3.95)	3 (1.57)
*Enterococcus faecalis*	21 (4.29)	69 (12.99)	84 (44.21)
Other enterococci	1 (0.20)	6 (1.12)	16 (8.42)
*Streptococcus *spp.	35 (7.15)	17 (3.20)	ND^a^
*Bifidobacterium *spp.	4 (0.81)	74 (13.93)	3 (1.58)
*Lactobacillus *spp.	2 (0.40)	6 (1.13)	15 (7.89)
Other Gram-positive bacteria^b^	22 (4.49)	31 (5.83)	ND
2. Gram-negative bacteria:			
*Acinetobacter johnsonii*	5 (1.03)	ND	ND
*Bacteroides *spp.	ND	6 (1.13)	2 (1.05)
*Burkholderia *spp.	2 (0.40)	5 (0.95)	ND
*Citrobacter *spp.	7 (1.44)	4 (0.75)	5 (2.63)
*Escherichia coli*	37 (7.56)	94 (17.70)	50 (26.32)
*Klebsiella *spp.	7 (1.44)	23 (4.33)	3 (1.58)
*Pantoea agglomerans*	ND	ND	2 (1.05)
*Enterobacter *spp.	7 (1.44)	30 (5.64)	4 (2.10)
Other Gram-negative bacteria^c^	11 (2.25)	5 (0.95)	ND

Total number of colonies identified	489	531	190

^a^ND, not detected; ^b^Other Gram-positive bacteria: *Actinomyces *spp., *Kocuria *spp., *Propionibacterium *spp.; ^c^Other Gram-negative bacteria: *Kluyvera cryocescens, Pseudomonas *spp., *Shigella *spp.

All the milk samples contained *S. epidermidis *and, in general, the percentage of samples in which staphylococci, enterococci and streptococci were isolated was more similar to that found among the feces of the breast-fed infants than to that achieved by feces of the formula-fed group (Table 5). A total of 489 breast milk isolates were randomly isolated and 304 (62.16%) were identified as *S. epidermidis *(Table 5). The rest of the bacterial groups found in feces of breast-fed infants were also detected in breast milk (Table 5). Streptococci were isolated from some samples of milk and feces of breast-fed infants but, in contrast, they could not detected in feces of the formula-fed ones (Table 5).

### Genotyping of the *S. epidermidis *isolates by Random Amplification of Polymorphic DNA (RAPD)

The 444 *S. epidermidis *isolates from feces of breast-fed infants and breast milk were genetically typified by the RAPD technique and the analysis of the profiles revealed the existence of 51 different genotypes. A representative of each RAPD profile was selected for further characterization. In addition, comparison of the RAPD profiles of fecal *S. epidermidis *with those obtained from breast milk isolates revealed that the same strain was shared by milk and infant feces in 12 of the 16 mother-infant pairs [see additional file 1].

### Characterization of the *S. epidermidis *strains

The 51 *S. epidermidis *strains were screened for the presence of potential virulence traits [see additional file 1]. In relation to adhesin-encoding genes, a multiplex PCR assay revealed the presence of the genes *embp *and *atlE *in all the strains. In contrast the *fbe *gene could be detected in only 13 strains (25%). The biofilm-related *icaD *gene was detected in 11 strains (20%) and, in general, there was a good correlation between the presence of such gene and the results obtained using the CRA assay, which determines potential for biofilm production. Hemolytic activity could not be detected among the assayed strains.

Among the 17 strains showing oxacillin resistance, the *mecA *gene could be detected by PCR in 9 (53%). In contrast, *mecA *amplification was obtained from 6 oxacillin-sensitive strain. Only 3 strains showed the simultaneous presence of *mecA *and *icaD *[see additional file 1]. The type of SCC *mec *was determined in all the *mec*A^+ ^strains. The *ccr*B gene could be amplified from all the *mec*A^+ ^strains and, on the basis of the *ccr*B restriction pattern with *Hinf*I (type IV: 264, 227 and 154 pb; type III: 537 and 106 bp) or with *Hinf*I/*Bsm*I (type IV: 227, 171, 153 and 93 bp; type III: 320, 174, 106 and 44 bp), all were assigned to type IV, which is generally carried by community-acquired staphylococci.

The determination of the MIC's of 21 antibiotics or antibiotics mixtures for the 51 *S. epidermidis *strains revealed that all of them were sensitive to the lower concentration of nitrofurantoin (32 μg/ml), vancomycin (≤ 2 μg/ml; with one exception) and rifampin (1 μg/ml; with two exceptions), while the results against the rest of antibiotics were variable depending on the strain [see additional file 2]. Independently of their origin, most of the strains were sensitive to quinupristin/dalfopriscin (≤ 0.25 μg/ml), trimethoprim/sulfamethoxazole (MIC < 2/38 μg/ml), gentamicyn (≤ 2 μg/ml), linezolid (≤ 2 μg/ml), fosfomycin (≤ 16 μg/ml), ciprofloxacin (≤ 0.5 μg/ml), chloramphenicol (≤ 16 μg/ml), ampicillin (≤ 4 μg/ml) and teicoplanin (≤ 1 μg/ml). The percentage of susceptible strains was lower for imipenem (≤ 0.12 μg/ml), penicillin (≤ 4 μg/ml), and tetracycline (≤ 8 μg/ml).

### Characterization of the *Enterococcus faecium *and *Enterococcus faecalis *strains

None of the 4 *E. faecium *strains tested have the presence of any virulence determinant (*ccf*, *cpd*, *cad*, *cob*, *efaA*_*fs*_, *efaA*_*fm*_, *agg*_2_, *gelE*,*cylA*, *eps*_*fs*_) while all the *E. faecalis *isolates tested possessed some potential virulence determinants [see additional file 3]. All the sex pheromone determinants were detected in 19 *E. faecalis *strains but the gene encoding cytolysin (*cylA*) couls only be detected in 7 strains. The results for the rest of the enterococcal genes were variable depending on the strains [see additional file 3].

All the *E. faecium *and *E. faecalis *strains were susceptible to low concentrations of penicillin, ampicillin, ciprofloxacin, fosfomycin, nitrofurantoine, tetraciclyne, erythromycin, vancomycin, teicoplanin, chloramphenicol and rifampicin [see additional file 4]. The percentage of strains resistant to quinupristin/dalfopriscin (MIC ≥ 4 μg/ml) was 79.31% while 34.48% of them showed resistance to streptomycin (MIC > 1000 μg/ml). Only one strain was resistant to gentamycin (MIC > 500 μg/ml) and only another one to linezolid (MIC ≥ 8 μg/ml) [see additional file 4].

## Discussion

Colostrum and milk play key roles in the initiation, development and composition of the infant gut microbiota since they contain a variety of factors, such as inmunoglobulins, inmunocompetent cells, fatty acids, polyamines, oligosaccharides, lysozyme, lactoferrin, and antimicrobial peptides, that modulate bacterial growth in the intestinal ecosystem. In addition, breast milk is an important and continuous source of commensal bacteria, including staphylococci, streptococci, and lactic acid bacteria, to the infant gut [8-10]. Therefore, it is not strange that the bacterial composition of the faecal flora of the breast-fed infant reflects the bacterial composition of breast milk [9].

In this work, *S. epidermidis *was the predominant species in milk of the lactating women and in the feces of their respective infants while it was almost absent in samples from feces of formula-fed infants. Previously, different studies have reported that this bacterial species is the predominant one in human milk from healthy women [9,10]. In contrast, *E. faecalis *was the dominant species among the isolates obtained from feces of formula-fed infants. Similarly, a molecular analysis revealed that *E. faecalis *was present in feces of a formula-fed infant on the sixth day of life but, in contrast, *S. epidermidis *could not be detected [13]. It has been suggested that the major differences between the microbiological composition of human milk and infant formula are probably the main factor responsible for the differences observed between the gut microbiota of breast- and formula-fed infants [1-4,14]. Other bacterial groups, such as lactobacilli were less prevalent and this fact may be due to their lower presence or to the fact that their isolation is difficult with the culture media used in this study.

Interestingly, the same *S. epidermidis *strain (as determined by genetic profiling) was isolated from milk and feces of several each mother-infant pairs. In the last years, it has been shown that breast milk plays an important role in the vertical mother-to-child transmission of lactic acid bacteria [5,8,15,16]. In this context, our results indicate that an abundant presence of *S. epidermidis *in the infant gut is a differential feature of the feces of breast-fed infants when compared to those of formula-fed infants.

Studies carried 20 years ago already described that staphylococci were common in feces of breast-fed infants [12,17,18]. More recently, it has been shown that coagulase-negative staphylococci colonized 100% of breast-fed Western infants from day 3 onwards [19]. Such staphylococci colonized vaginally and cesarean section-delivered infants equally early. Some authors suggest that, in fact, staphylococcal colonization of the infant gut has increased from the 70s to the present [19,20]. It has been speculated that this may be an effect of a highly hygienic lifestyle which leads to a delayed acquisition of "traditional" fecal bacteria, such as enterobacteria [19]. In their absence, staphylococci become the first gut colonizers and the results of our work suggest that breast milk could be the main source. Then, the population decreased significantly from 4 week until 6 month of age. Similarly, in our study the number of samples from breast-fed infants in which *S. epidermidis *could be isolated decreased from week 1 to week 5.

The different *S. epidermidis *strains isolated from feces of the breast-fed infants were submitted to a characterization scheme that included the detection of virulence-associated determinants and the profile of antibiotic resistances in order to confirm the prevalence of non-pathogenic isolates in the healthy infant gut. Among the *S. epidermidis *strains analyzed, the presence of adhesion-related genes was very high, independently of the sample from which they were isolated. All of them carried the *embp *and *atlE *genes and 25% of the strains harbored the *fbe *gene. The cell surface proteins may help to explain the high prevalence of *S. epidermidis *in breast milk since they could contribute to the bacterial attachment to the mammary areola and ducts throughout the lactation period. In contrast, the percentage of strains carrying the biofilm-related *ica *operon was much lower (20%). A potential relationship between *S. epidermidis *infection and the presence of such operon has been reported [21]. In fact, biofilm formation has been described in many cases of staphylococcal mastitis and this is the reason why such property is considered as a potential virulence factor [22]. A few strains showed methicillin resistance but methicillin-resistant staphylococci are being reported with increasing frequency in the community and they are commonly isolated from healthy hosts [23]. Globally, most of the *S. epidermidis *strains characterized in this study harbour several adhesion factors but not antibiotic resistance or virulence determinants. Since staphylococcal strains provided first by colostrum and, later, by breast milk may successfully compete with potentially pathogenic strains found in the hospital environment, their application as probiotics in neonatal units could be considered in the future if works in progress (including complete genome sequencing and analysis) confirm the safety of selected strains.

Enterococci, and particularly *E. faecium *and *E. faecalis*, become normal components of the human gastro-intestinal soon after birth. On the other hand, enterococci are opportunistic pathogens that may cause nosocomial infections in neonates suffering underlying diseases [24]. However, the presence of virulence determinants and the antibiotic resistance pattern appears to be strain-specific among isolates studied so far [25,26]. In fact, it seems that human isolates involved in clinical infection fell into a well defined subgroup, which suggest that there may be a genetic basis for strains associated with human disease [27,28]. In addition, enterococci not involved in human clinical infection are generally sensitive to clinically relevant antibiotics, including vancomycin [24,26], as happened with the enterococcal strains analyzed in this work.

## Conclusion

Our results indicate that the feeding practice (breast- or formula fedding) had a significant effect on bacterial counts and fecal microbiota composition. *S. epidermidis *is the most prevalent species in feces of breast-fed infants while it is practically absent in those of formula fed-infants. Therefore, *S. epidermidis *can be considered as a differential trait of the fecal microbiota of breast-fed infants although this finding requires further confirmation in larger studies. The staphyloccal isolates only contain a low number of virulence determinants and are sensitive to most of the antibiotics tested. Additionally, we have observed that *E. faecalis *is the second bacterial species in feces of the breast-fed group but it is also present in all the samples from the formula-fed one. The characterization of several representative enterococcal isolates revealed that none of them were resistant to vancomycin. Streptococci were isolated from some samples of milk and feces of breast-fed infants but, in contrast, they could not detected in feces of the formula-fed ones.

## Methods

### Subjects and sampling

A total of 23 women and their respective infants participated in the study and they were enrolled according with the following criteria: (a) healthy women without present or past underlying conditions; (b) normal full-term pregnancy; (c) vaginal delivery; and (d) absence of infant and/or maternal perinatal problems, including mastitis. Among the 23 women, 16 breast-fed their infants while the remaining 7 voluntarily choose a commercial formula devoid of prebiotics to fed their infants despite they were advised of the benefits of breastfeeding. The assessment of sample size for both groups of infants (breast-fed and formula-fed) was based on an estimated large effect size of the feeding practice on the infant gut microbiota (0.8 log units of diference in the mean values of bacterial counts), with an α at 0.05 and a statistical power of 83% in a variance analysis of repeated measures. All volunteers gave written informed consent to the protocol, which was approved by the Ethical Committee of Hospital Joan XXIII (Tarragona, Spain). The participants provided a sample of breast milk (breastfeeding group) and infant feces at days 7, 14, and 35 after birth. All the samples were collected in sterile tubes as previously described [10] and kept at 4°C until delivery to the laboratory.

### Isolation and enumeration of bacteria

Proper peptone water dilutions of the milk and feces samples were plated in triplicate onto Brain Heart Infusion (BHI, Oxoid, Basingstoke, UK; a general-purpose medium suitable for the cultivation of non-fastidious bacteria, yeasts and moulds), Violet Red Bile Agar (VRBA; Difco, Detroit, MI; a selective medium for the isolation of enterobacteria) and Columbia Nadilixic Acid Agar (CNA, BioMerieux; a highly nutritious, general-purpose medium for the isolation and cultivation of fastidious microorganisms) agar plates, which were aerobically incubated at 37°C for 24 h. Parallel, the same samples were also cultured on Wilkins-Chalgren (WCh, Oxoid; a general medium for isolating anaerobic bacteria) and de Man, Rogosa, and Sharpe (MRS, Oxoid; a medium for the isolation of lactic acid bacteria and bifidobacteria) agar plates, which were incubated anaerobically (85% nitrogen, 10% hydrogen, 5% carbon dioxide) in an anaerobic workstation (MINI-MACS, DW Scientific, Shipley, UK) at 37°C for 48 h. Between 5–10 isolates from each culture medium where growth was observed (~35 isolates per sample and week) were randomly selected, grown in BHI broth and stored at -80°C in the presence of glycerol (30%, v/v).

### Identification of the bacterial isolates

The selected isolates were observed by optical microscopy to determine their morphology and Gram staining. Additionally, they were tested for catalase, oxidase and coagulase activities and for grow on plates of Baird-Parker (BP, BioMerieux) and Kanamycin Aesculin Azide Agar (KAA, Oxoid). All the isolates corresponding to samples (milk/feces) obtained at weeks 1, 2 and 5 were identified to the species level. Initially, most of the isolates that, on the basis of such preliminary tests, seemed to belong to the genus *Staphylococcus *were identified as *S. epidermidis, S. aureus *or *S. hominis *by a novel multiplex PCR method based on the *dnaJ *genes. Briefly, a single colony growing on solid media was removed with a sterile plastic tip and resuspended in 100 μl of sterile deionized water in a microcentrifuge tube. Then 100 μl of chloroform/isoamyl alcohol (24:1) was added to the suspensions, and after vortexing for 5 s the mixture was centrifuged at 16,000 × g for 5 min at 4°C. Then 5–10 μl of the upper aqueous phase was used as a source of DNA template for PCR with primers J-StGen (5'-TGGCCAAAAGAGACTATTATGA-3'), J-StAur (5'-GGATCTCTTTGTCTGCCG-3'), J-StEpi (5'-CCACCAAAGCCTTGACTT-3') and J-StHom (5'-TTGACCACTACCCTCACAC-3') in a Icycler thermocycler (Bio-Rad Laboratories, Richmond, CA). The primer pairs J-StGen/J-StAur, J-StGen/J-StEpi and J-StGen/J-StHom result in a 337 bp *S. aureus *species-specific fragment, 249 bp *S. epidermidis *species-specific fragment and a 589 bp *S. hominis *species-specific fragment, respectively. PCR condicions were as follows: 1 cycle of 94°C for 4 min, 30 cycles of 94°C for 30 sec, 60°C for 30 sec, and 72°C for 30 sec, and a final extension of 72°C for 5 min. On the other hand, most of the isolates that seemed to belong to the genus *Enterococcus *could be identified by PCR species-specific detection of enterococcal *ddl *genes, which encode D-alanine:D-alanine ligases, following the protocol described by Dutka-Malen et al. [29]. Confirmation of staphylococci and enterococci identification and identification of the rest of the isolates was performed by PCR sequencing of a 470 pb fragment of the 16S rRNA gene as described by Kullen et al. [30]. The amplicons were purified using the Nucleospin^® ^Extract II kit (Macherey-Nagel, Düren, Germany) and sequenced at the Genomics Unit of the Universidad Complutense de Madrid, Spain. The resulting sequences were used to search sequences deposited in the EMBL database using BLAST algorithm and the identity of the isolates was determined on the basis of the highest scores (> 98%).

### Genotyping of the *S. epidermidis*, *E. faecium *and *E. faecalis *isolates by Random Amplification of Polymorphic DNA (RAPD)

The *S. epidermidis, E. faecium *and *E. faecalis *isolates were typified by RAPD. DNA was extracted from isolated colonies following the protocol of Ruiz-Barba et al. [31] and was used as a template to determine the RAPD profile with the primer OPL5 (5'-ACGCAGGCAC-3'') [32]. This technique was also used to compare *S. epidermidis *isolates obtained from breast milk and infant feces of the different mother-infant pairs in order to know if there may be a vertical mother-to-child transmission of this species.

### Screening for potential virulence determinants, *mecA, SSCmec *and antibiotic susceptibility among the *S. epidermidis *isolates

Based on their different RAPD, 51 *S. epidermidis *strains were selected for further studies. Presence of genes *embp*, *fbe *and *atlE *(which products are involved in adhesion) and *icaD *(involved in biofilm formation), was evaluated using primers couples described previously [33-36]. In the case of *fbe*, *atlE *and *icaD*, a novel multiplex PCR format was designed using the following conditions: 5 min at 94°C followed by 30 cycles of 94°C for 1 min, 60°C for 30 s, 72°C for 1 min and, then, a final extension of 5 min at 72°C. On the other hand, conditions for amplification of the *embp *gene were as follows: 5 min at 94°C for 1 cycle followed by 30 cycles of 30 s at 94°C, 1 min at 58°C, 1 min at 72°C, and a final extension step at 72°C for 5 min. The hemolytic activity of the isolates was determined on Columbia agar supplemented with 5% horse blood (COH, BioMerieux). After an incubation of 72 h at 37°C, the plates were analyzed and the isolates were classified as non hemolytic (no halo), moderately hemolytic (halo < 1.5 mm) or strongly hemolytic (halo > 1.5 mm). The ability of the *S. epidermidis *isolates to form biofilms was assessed using the Congo Red agar assay (CRA) [37]. Presence of the *mecA *gene, conferring methicillin-resistance, was evaluated in *S. epidermidis *isolates following the protocol described in a previous study [38]. The *mecA *gene is located in a mobile element in the staphylococcal chromosome, constituting the cassette SCC *mec*. The SCC *mec *element was submitted to a typing procedure previously described [39], which is based on the PCR amplification of the *ccr*B gene followed by a restriction fragment length polymorphism (RFLP) analysis using endonucleases *Hinf*I and *Bsm*I.

The determination of the MICs to several antibiotics was evaluated by a microdilution method using the Sensititre plates Staenc1F (Trek Diagnostic Systems, Cleveland, OH) following the manufacturer's instructions. The antibiotics analyzed were: penicillin, ampicillin, amoxycillin-clavulanic acid, teicoplanin, chloramphenicol, erythromycin, mupirocin, streptomycin, gentamicin, clindamycin, oxacillin, ciprofloxacin, fosfomycin, imipenem, nitrofurantoine, trimethoprim-sufamethoxazole, tetracycline, vancomycin, linezolid, quinupristin-dalfopriscin and rifampin.

### Screening for potential virulence determinants, *van *genes and antibiotic susceptibility among the enterococcal isolates

Similarly, 25 *E. faecalis *and 4 *E. faecium *strains (one representative of each of the RAPD group found in this study) were further characterized. A novel multiplex PCR method was used to detect the presence of virulence determinants encoding sex pheromones (*ccf*, *cpd*, *cad*, *cob*), adhesins (*efaA*_*fs*_, *efaA*_*fm*_) and products involved in aggregation (*agg*_*2*_), biosynthesis of an extracellular metalloendopeptidase (*gelE*), biosynthesis of cytolysin (*cylA*) and immune evasion (*eps*_*fs*_). The primers couples and PCR conditions used to detect all the genes cited above were those proposed by Eaton and Gasson [25]. Control strains used in PCR experiments were *E. faecalis *strains F4 (*efaA*_*fs*_^+ ^*gelE*^+ ^*agg*^+ ^*cylMBA*^+ ^*esp*^+ ^*cpd*^+ ^*cob*^+ ^*ccf*^+ ^*cad*^+^), P36 (*efaA*_*fs*_^+ ^*gelE*^+ ^*agg*^+ ^*cylA*^+ ^*esp*^+ ^*cpd*^+ ^*cob*^+ ^*ccf*^+ ^*cad*^+^) and P4 (*efaA*_*fs*_^+ ^*gelE*^+ ^*agg*^+ ^*cylA*^+ ^*cpd*^+ ^*cob*^+ ^*ccf*^+ ^*cad*^+^), and *E. faecium *P61 (*efaA*_*fm*_^+ ^*esp*^+^) [25]. The hemolytic activity of the isolates and their ability to form biofilms were assessed exactly as described for the staphylococcal isolates.

PCR reactions for *vanA *and *vanB *genes were prepared as described Dutka-Malen et al. [29] and Ramos-Trujillo et al. [40], respectively. *E. faecium *BM4147 (resistant to vancomycin, VanA^+^) and *E. faecalis *V583 (resistant to vancomycin, VanB^+^) were used as positive controls. Detection of *vanD*, *vanE *and *vanG *genes in the *E. faecalis *isolates was performed as previously described [41-43]. The determination of the MICs to several antibiotics was evaluated by the microdilution method cited for the staphylococcal isolates.

### Statistical analysis

Microbiological data, recorded as colony forming units (CFU) per gram of feces, or milliliter of milk, were transformed to logarithmic values before statistical analysis. The reported values of bacterial counts are the mean values of duplicate or triplicate determinations and the standard deviation (SD) of the mean. Bacterial counts in feces samples were analyzed by two-way ANOVA for repeated measures using the General Lineal Model procedure to determine the effect of feeding practice (breast feeding and formula feeding) and infant age (days 7, 14, and 35 after birth) or culture media used for enumeration of bacterial counts (BHI, VRBA, CNA, WCh, and MRS) and their interaction. The two-way ANOVA was followed by Duncan's multiple range tests (α = 0.05). A one-way repeated measures ANOVA was used to test for differences amongst the means of bacterial counts from breast milk for each culture media used with sampling occasion as repeated measures variable. The statistical software package SAS version 9.1 (SAS Institute Inc., Cary, NC, USA) was used for these analysis.

Student's *t*-tests were applied to determine statistically significant differences between the bacterial counts in feces of breast-fed and formula-fed infants at each infant age and for each culture media used for enumeration of bacterial counts. Comparison of bacterial counts in breast milk obtained at different sampling times (1, 2, and 5 weeks) was made also using Student's *t*-tests. Two-sided probability (P) values ≥ 0.05 were considered non significant. Identified bacterial isolates from infant feces were analyzed to evaluate the association between the presence of a specific kind of bacteria in the fecal samples and the feeding practice using a χ^2 ^tests and a significance level of P < 0.05.

The Pearson's product-moment correlation was used to assess the association between bacterial counts in breast milk and in infant feces for each medium and sampling time. Student's *t*-tests, χ^2 ^tests and Pearson's test were carried out with the Statgraphics Plus 5.0 software (Manugistics, Inc., Rockville, Md.).

## Authors' contributions

EJ and SD carried out the microbiological analysis of the samples, AM designed the primers and PCR conditions. RA assisted in the preparation of material and participated in the identification of the isolates. MA and NG collected the samples and the epidemiological data. MJ and LF participated in the design of the study and performed the statistical analysis. AG coordinated the enrolment of the volunteers and the collection of the samples, participated in the design of the study and revised the manuscript. JMR conceived of the study, coordinated it and drafted the manuscript. All authors read and approved the final manuscript.

## Supplementary Material

Additional file 1 Analysis of the *S. epidermidis *strains. A table showing the presence of potential virulence determinants and other traits among the 51 *S. epidermidis *strains. Click here for file

Additional file 2 Resistance to different antibiotics among the *S. epidermidis *strains. A table showing MIC distribution and percentage of resistance to different antibiotics among the *S. epidermidis *strains. Click here for file

Additional file 3 Analysis of the enterococcal strains. A table showing the presence of potential virulence determinants and other traits among the enterococcal strains. Click here for file

Additional file 4 Resistance to different antibiotics among the enterococcal strains. A table showing MIC distribution and percentage of resistance to different antibiotics among the enterococcal strains. Click here for file
