# Supplementary material for: Staphylococcus epidermidis: A differential trait of the fecal microbiota of breast-fed infants
Source: BMC Microbiol. 2008 Sep 10;8:143. doi: 10.1186/1471-2180-8-143 (PMC2551609; doi:10.1186/1471-2180-8-143)
Supplement: Additional file 1 — Analysis of the S. epidermidis strains. A table showing the presence of potential virulence determinants and other traits among the 51 S. epidermidis strains. [file 1471-2180-8-143-S1.pdf]

Analysis of virulence determinants among the 51 *S. epidermidis* strains.

Mother-infant Pair	Strain	Source[a]	*mecA*	SCC type	*fbe*	*atlE*	*icaD*	*embp*	CRA[b]	Oxacillin resistance	Hemolysin
1	LC002	M/BF	+	IV	-	+	+	+	+	+	-
1	LC011	M/BF	+	IV	-	+	-	+	-	+	-
1	LC015	M	-	-	+	+	-	+	-	-	-
1	LC016	M	-	-	-	+	-	+	-	-	-
1	LC017	M	-	-	-	+	-	+	-	-	-
1	LC019	M	-	-	+	+	-	+	-	-	-
1	LC044	M	+	IV	+	+	-	+	-	+	-
1	LC047	M	-	-	+	+	-	+	-	+	-
2	LE010	M	-	-	-	+	-	+	-	-	-
2	LE011	M	-	-	-	+	-	+	-	-	-
2	LE035	M	-	-	-	+	-	+	-	-	-
3	LF001	M/BF	-	-	-	+	+	+	+	-	-
3	LF003	M	-	-	-	+	-	+	-	+	-
4	LG005	M/BF	-	-	+	+	-	+	-	-	+
4	LG006	M	+	IV	-	+	-	+	-	-	-
4	LG011	M/BF	-	-	-	+	-	+	-	+	-
4	LG5021	M	-	-	+	+	-	+	-	-	-
4	LG5022	M	-	-	+	+	-	+	-	-	-
4	LG5023	M/BF	-	-	+	+	+	+	-	-	-
5	LH522	M	-	-	-	+	-	+	-	+	-
5	LH524	M	-	-	-	+	-	+	-	-	-
6	LI55	M	-	-	-	+	-	+	-	-	-
7	LO102	M/BF	-	-	-	+	-	+	-	+	-
7	LO103	M/BF	-	-	-	+	-	+	-	-	-
7	LO502	M	-	-	-	+	+	+	-	-	-
7	HO122	BF	+	IV	+	+	-	+	-	-	-
8	LP222	M	+	IV	+	+	-	+	-	-	-
8	LP223	M/BF	+	IV	+	+	-	+	-	+	-
8	LP242	M/BF	-	-	-	+	-	+	-	-	-
8	LP245	M	+	IV	-	+	+	+	+	-	-
9	LM102	M/BF	-	-	-	+	-	+	-	-	-
9	LM141	M	-	-	-	+	-	+	-	+	-
9	LM152	M	-	-	-	+	-	+	-	-	-
10	LV104	M	-	-	-	+	+	+	+	-	-
10	LV221	M	+	IV	+	+	-	+	-	-	-
10	LV222	M	+	IV	-	+	-	+	-	+	-
10	LV521	M	+	IV	+	+	-	+	-	+	-
11	LX103	M/BF	-	-	-	+	-	+	+	-	-
11	LX123	M/BF	+	IV	-	+	+	+	+	+	-
11	LX121	M	-	-	-	+	-	+	-	-	-
11	LX154	M	+	IV	-	+	-	+	-	-	-
12	LZ153	M/BF	+	IV	-	+	-	+	-	+	-

Analysis of virulence determinants among the 51 *S. epidermidis* strains (cont.)

Mother-infant Pair	Strain	Source[a]	*mecA*	SCC type	*fbe*	*atlE*	*icaD*	*embp*	CRA[b]	Oxacillin resistance	Hemolysin
12	LZ221	M/BF	-	-	-	+	-	+	-	-	-
12	HZ242	BF	-	-	-	+	+	+	-	+	-
13	LCC141	M/BF	+	IV	-	+	-	+	-	+	-
13	LCC254	M	-	-	-	-	-	+	-	+	-
13	LCC521	M	-	-	-	+	+	+	+	-	-
14	LGG152	M/BF	-	-	-	+	-	+	-	-	-
14	LGG252	M	-	-	-	+	-	+	-	-	-
15	LDD121	M/BF	-	-	-	+	+	+	+	-	-
16	LFF101	M/BF	-	-	-	+	-	+	-	-	-

[a]Source: isolated from milk (M), feces of breast-fed infants (BF) or both sources within the same mother-infant pair (M/BF); [b]CRA: Congo Red Agar Assay
