# Supplementary material for: Staphylococcus epidermidis: A differential trait of the fecal microbiota of breast-fed infants
Source: BMC Microbiol. 2008 Sep 10;8:143. doi: 10.1186/1471-2180-8-143 (PMC2551609; doi:10.1186/1471-2180-8-143)
Supplement: Additional file 2 — Resistance to different antibiotics among the S. epidermidis strains. A table showing MIC distribution and percentage of resistance to different antibiotics among the S. epidermidis strains. [file 1471-2180-8-143-S2.pdf]

MIC distribution and percentage of resistance to different antibiotics among the *S. epidermidis* strains.

Antibiotic	Number of isolates with MIC (μg/ml) of																	S/R	% Resistance
	**≤0.03**	**0.12**	**0.25**	**0.5**	**1**	**2**	**4**	**8**	**16**	**32**	**64**	**100**	**125**	**128**	**256**	**500**	**≤1000**		
Penicillin	8	5		6	3	6	5	18										≤0.12/>4	35.29
Ampicillin		13	6	4	6	7	2	6				7						≤0.12/>4	25.49
Oxacillin			12	14	3	5	17[c]											≤2/>2	33.33
Ciprofloxacin			29	15	5	2												≤1/>1	3.91
Fosfomycin									45	4	1		1					≤16/>16	11.76
Nitrofurantoine										51								≤32/≥128	0
Mupirocine							39								12			≤8/>256	0
Streptomycin																	51	≤1000/>1000	0
Gentamycin						45	4							1		1		≤1/>2	11.76
Linezolid			3	3	16	20	8	1										≤4/≥4	1.96
Tetracycline							32	19										≤4/>8	0
Erythromycin			26	4	1	2	18											≤0.5/>4	0
Clindamycin				40	3	8												≤0.5/>2	15.68
Vancomycin				1	18	30	1	1										≤4/>8	0
Teicoplanin				21	16	11	3											≤4/>8	0
Q/D[a]			37	8	3		3											≤2/>2	5.88
Chloranphenicol								40	11									≤8/>8	21.57
Rifampin					49	2												-	-
Imipenem		34	6	2	5	3	1											-	-
	**≤1/38**	**≥2/38**																	
T/S[b]	37	14																-	-
	**≤0.5/0.25**	**1/0.5**	**2/1**	**4/2**	**>8/4**														
Augmentine	33	10	4	2	1													≤0.5/0.25//>2/1	5.88

[a]Q/D: Quinupristin/Dalfopristin; [b]T/S: Trimethoprim/sulfamethoxazole; [c]17 isolates >2 μg/ml of Oxacillin.
