# Supplementary material for: Staphylococcus epidermidis: A differential trait of the fecal microbiota of breast-fed infants
Source: BMC Microbiol. 2008 Sep 10;8:143. doi: 10.1186/1471-2180-8-143 (PMC2551609; doi:10.1186/1471-2180-8-143)
Supplement: Additional file 3 — Analysis of the enterococcal strains. A table showing the presence of potential virulence determinants and other traits among the enterococcal strains. [file 1471-2180-8-143-S3.pdf]

Analysis of virulence determinants among the enterococcal strains

Mother/infant pair	Strain	Source[a]	*gelE*	*cylA*	$efaA_{fs}$	*ccf*	*cad*	*cob*	*cpd*	*eps*	*agg*	*vanA*	*vanB*	*vanD*	*vanE*	*vanG*	Hemolysis
	E. faecalis:																
1	HC046	BF	+	-	+	+	+	+	+	-	-	-	-	-	-	-	-
2	HE10	BF	+	-	+	+	-	-	+	+	-	-	-	-	-	-	-
3	MF002	M/BF	+	+	+	+	+	+	+	-	+	-	-	-	-	-	+
4	HG502	BF	-	-	+	+	+	+	+	+	+	-	-	-	-	-	-
5	HI04	BF	+	-	+	+	+	+	+	+	+	-	-	-	-	-	-
7	MO22	BF	+	-	+	+	-	+	+	-	-	-	-	-	-	-	-
8	HP523	BF	+	+	+	+	-	-	+	+	+	-	-	-	-	-	-
9	HM501	BF	-	-	+	-	+	+	+	+	-	-	-	-	-	-	-
10	MV02	BF	+	-	+	+	+	+	+	+	+	-	-	-	-	-	-
11	MX01	BF	+	-	+	+	+	+	+	-	-	-	-	-	-	-	-
11	HX152	BF	+	-	+	+	+	+	+	-	-	-	-	-	-	-	-
12	MZ02	BF	-	-	+	-	+	+	+	-	+	-	-	-	-	-	+
12	HZ241	BF	+	+	+	+	+	+	+	-	+	-	-	-	-	-	-
13	MCC02	BF	+	+	+	+	+	+	+	+	+	-	-	-	-	-	-
13	HCC523	BF	+	-	+	+	+	+	+	-	+	-	-	-	-	-	-
15	MDD01	BF	+	-	+	+	+	+	+	-	+	-	-	-	-	-	+
17	MA006	FF	+	-	+	+	+	+	+	+	+	-	-	-	-	-	-
18	MK01	FF	+	-	+	+	+	+	+	+	-	-	-	-	-	-	-
18	HK103	FF	+	-	+	+	-	-	+	-	-	-	-	-	-	-	-
19	MU23	FF	+	+	+	+	+	+	+	-	+	-	-	-	-	-	+
20	MW02	FF	+	-	+	+	+	+	+	+	+	-	-	-	-	-	+
20	HW121	FF	-	+	+	+	+	+	+	+	+	-	-	-	-	-	-
21	HBB141	FF	+	+	+	+	+	+	+	+	+	-	-	-	-	-	-
21	HBB501	FF	+	-	+	+	+	+	+	+	+	-	-	-	-	-	+

Analysis of virulence determinants among the enterococcal strains (cont.)

Mother/infant pair	Strain	Source	*gelE*	*cylA*	$efaA_{fs}$	*ccf*	*cad*	*cob*	*cpd*	*eps*	*agg*	*vanA*	*vanB*	*vanD*	*vanE*	*vanG*	Hemolysis
	E. faecium:																
5	LH552	M/BF	-	-	-	-	-	-	-	-	-	-	-	-	-	-	-
17	HA006	FF	-	-	-	-	-	-	-	-	-	-	-	-	-	-	-
18	HK522	FF	-	-	-	-	-	-	-	-	-	-	-	-	-	-	-
20	HW521	FF	-	-	-	-	-	-	-	-	-	-	-	-	-	-	-
