# Supplementary material for: Staphylococcus epidermidis: A differential trait of the fecal microbiota of breast-fed infants
Source: BMC Microbiol. 2008 Sep 10;8:143. doi: 10.1186/1471-2180-8-143 (PMC2551609; doi:10.1186/1471-2180-8-143)
Supplement: Additional file 4 — Resistance to different antibiotics among the enterococcal strains. A table showing MIC distribution and percentage of resistance to different antibiotics among the enterococcal strains. [file 1471-2180-8-143-S4.pdf]

MIC distribution and percentage of resistance to different antibiotics among the enterococcal strains

	Number of isolates with a MIC (µl/ml) of															
**Antibiotic**	**≤0.25**	**0.5**	**1**	**2**	**4**	**8**	**16**	**32**	**64**	**128**	**256**	**>500**	**≤1000**	**>1000**	**S/R[a]**	**% Resistance**
Penicillin				18	10	1									≤8/≥16	0
Ampicillin		1	23	4	1										≤8/≥16	0
Oxacillin					29[d]										-	-
Ciprofloxacin		1	14	14											≤1/≥4	0
Fosfomycin							1	20	8						≤64/≥256	0
Nitrofurantoine								24	5						≤32/≥128	0
Mupirocine					4						25				-	-
Streptomycin													19	10	≤1000/>1000	34.48
Gentamycin					1	12				15		1			≤500/>500	3.44
Linezolid				22	6	1									≤2/≥8	3.44
Tetracycline					13	16									≤4/≥16	0
Erythromycin	3	4	5	6	11										≤0.5/≥8	0
Clindamycin		1	1	27												
Vancomycin		2	7	14	6										≤4/≥32	0
Teicoplanin		29													≤8/≥32	0
Q/D[b]		2		4	23										≤1/≥4	79.31
Chloranphenicol						24	5								≤8/≥32	0
Rifampin			14	15											≤1/≥4	0
Imipenem			4	19	5	1									-	-

	**≤1/38**	**≥2/38**	S/R	% Resistance
T/S[c]	27	2	-	-

	**≤0.5/0.25**	**1/0.5**	**2/1**	**4/2**	**>8/4**	S/R	% Resistance
Augmentine	12	15	2			-	-

[a] S/R: Susceptible/Resistant [44], [b]Q/D: Quinupristin/Dalfopristin, [c]T/S: Trimethoprim/sulfamethoxazole; [d]29 isolates>2 µg/ml of Oxacillin
